# Timing Is Everything: International Variations in Historical Sexual Partnership Concurrency and HIV Prevalence

**DOI:** 10.1371/journal.pone.0014092

**Published:** 2010-11-24

**Authors:** Martina Morris, Helen Epstein, Maria Wawer

**Affiliations:** 1 Departments of Statistics and Sociology, University of Washington, Seattle, Washington, United States of America; 2 New York, New York, United States of America; 3 Population, Family and Reproductive Health, Johns Hopkins Bloomberg School of Public Health, Baltimore, Maryland, United States of America; Stanford University, United States of America

## Abstract

**Background:**

Higher prevalence of concurrent partnerships is one hypothesis for the severity of the HIV epidemic in the countries of Southern Africa. But measures of the prevalence of concurrency alone do not adequately capture the impact concurrency will have on transmission dynamics. The importance of overlap duration and coital exposure are examined here.

**Methodology/Principal Findings:**

We conducted a comparison of data from three studies of sexual behavior carried out in the early 1990s in Uganda, Thailand and the US. Using cumulative concurrency measures, the three countries appeared somewhat similar. Over 50% of both Thai and Ugandan men reported a concurrency within the last three partnerships and over 20% reported a concurrency in the last year, the corresponding rates among US men were nearly 20% for Blacks and Hispanics, and about 10% for other racial/ethnic groups. Concurrency measures that were more sensitive to overlap duration, however, showed large differences. The point prevalence of concurrency on the day of interview was over 10% among Ugandan men compared to 1% for Thai men. Ugandan concurrencies were much longer duration – a median of about two years – than either the Thai (1 day) or US concurrencies (4–9 months across all groups), and involved 5–10 times more coital risk exposure with the less frequent partner. In the US, Blacks and Hispanics reported higher prevalence, longer duration and greater coital exposure than Whites, but were lower than Ugandans on nearly every measure. Together, the differences in the prevalence, duration and coital exposure of concurrent partnerships observed align with the HIV prevalence differentials seen in these populations at the time the data were collected.

**Conclusions/Significance:**

There were substantial variations in the patterns of concurrent partnerships within and between populations. More long-term overlapping partnerships, with regular coital exposure, were found in populations with greater HIV epidemic severity.

## Introduction

The prevalence of HIV infection varies widely around the world. The systematic variations in prevalence trajectories have been evident for nearly 20 years, giving rise to the familiar classification of a country's epidemic as “concentrated” or “generalized.” The determinants of these differences remain a topic of much discussion and debate. While many studies find that the lifetime number of sexual partners is an important risk factor for HIV infection at the individual level, the population average lifetime number of partners by country do not correlate well with national HIV prevalence. [1], [2]. Similarly, while male circumcision has been shown to reduce HIV transmission at the individual level, and widespread male circumcision almost certainly helps explain why HIV rates are low in some parts of the world, this cannot explain why HIV rates among heterosexual men and women are ten to one hundred times higher in some African countries than they are in much of Europe, Asia and Latin America, where male circumcision is similarly uncommon. Rates of condom use and age of sexual debut also cannot explain international variations in HIV prevalence [3].

Because HIV is sexually transmitted, it is reasonable to assume that patterns of sexual behavior influence the scale of the epidemic in affected countries. However, if obvious factors such as numbers of sexual partners, male circumcision, age of sexual debut and rates of condom use don't fully explain these variations, what else contributes to the observed HIV rates across regions? This question is of more than academic interest, as the answer should guide HIV prevention efforts [4].

More than 15 years ago, a series of papers proposed that long term concurrent—or overlapping–partnerships might help explain the particularly high HIV infection rates seen in some African populations [5], [6], [7], [8]. Since then, computer modeling studies have shown that, *ceteris paribus*, HIV will spread more rapidly through a sexual network in which concurrency is common, than through one in which serial monogamy is exclusively practiced, even when overall partnership numbers are the same in both cases [9]. Data-driven simulation studies have also demonstrated that concurrency appears to be a driver of both the epidemics in South Africa [10] and the racial disparities in HIV prevalence in the United States [11], as well as a necessary factor for epidemic persistence in Zimbabwe [12]. The theory that concurrent sexual partnerships can accelerate the spread of HIV in populations where rates of partner change are otherwise relatively low is now generally accepted.

Empirical evidence for the role of concurrency in driving the spread of HIV generally falls into two categories: individual and population level studies. Here there is quite a bit confusion in the literature, due to the fact that traditional epidemiological study designs and methods cannot be used to identify the effects of concurrency. The misinterpretation of these empirical relationships lies behind much of the current “debate” over the significance of concurrency [13], [14], [15], [16], [17].

Individual level studies in epidemiology typically focus on the risk to a person enrolled in the study (the “index case”). But in the case of concurrency, the important effects operate asymmetrically in the context of a partnership. In partnerships, there are two types of risk – of acquiring infection and transmitting infection – and two types of individuals – the person who practices concurrency and the partners of that person. Concurrency theory predicts that concurrency increases the risk of transmission from the person who practices it, and it raises the risk of acquisition to the partners of that person. If the index case practices concurrency, their risk is, *ceteris paribus*, increased simply by the number of partners they have, not by the concurrency per se [18]. Thus the predicted empirical signature of concurrency's effect on transmission is not a correlation between index case concurrency and their own HIV status, but a correlation between index case concurrency and their partner's HIV status. Estimating this effect requires a study design that enrolls both partners in a sexual relationship; a design that is difficult to implement (especially for non-cohabiting partners) and rarely attempted.

Unfortunately, many studies have been published [19], [20], [21], presented at international meetings [22], or published as technical reports on in-house websites [23] that make this mistake. They operationalize the “concurrency effect” as a test of whether concurrency practiced by an index case predicts their HIV status, and misinterpret a negative finding as evidence disconfirming the concurrency hypothesis. This is a test of the wrong relationship, but these studies are repeatedly cited as empirical support by critics of the concurrency hypothesis.

Similar care needs to be exercised when evaluating the findings from population level studies. Here, it is important to bear in mind that HIV prevalence reflects incident cases that have accumulated over time, while most concurrency measurement captures more recent behavior, which may have changed over time. This makes traditional ecological analysis – correlation of concurrency prevalence to HIV prevalence at the population level – inappropriate. Several empirical studies that have sought to establish whether concurrency is associated with HIV at the population level have failed to recognize this [20], [23], and these have become the other contribution to the widely cited “negative” findings that have in turn generated debate in the current literature.

In studies with appropriate methodology, i.e., those that have collected data on partner's status and/or behavior, and HIV biomarkers, the findings have consistently supported the concurrency hypothesis [24], [25], [26], [27], [28]. This is also true for studies of other STIs [29], [30].

Another factor that complicates the current empirical findings, however, is that concurrency, like sexual partnerships generally, can take various forms, and these variations have important implications for the structure and connectivity of the resulting sexual networks [31], [32]. Concurrency can be as short as a single episode of sex with a secondary partner during an ongoing relationship with a primary partner, or it can go on for years, if one person has regular sexual contact with two or more people. Both forms involve “going back to” a previous partner, and starting a new partnership before the previous one has ended, the two signature features of concurrency that influence the reachable path and speed of HIV transmission. However, long term concurrency can be expected to increase epidemic potential more than short term concurrencies for three reasons. First, as with any infectious disease, duration of exposure itself increases risk. Short term concurrencies involve relatively fewer coital acts, lowering the risk of transmission. Second, when enough people are conducting overlapping relationships of long duration (and among heterosexuals, as long as both sexes have some prevalence of concurrency), this gives rise to a stable connected sexual network that provides consistent opportunities for ongoing transmission even when rates of partner change are low. Third, this “network” effect enhances the impact of the high transmission probability associated with the peak in viral load immediately following infection with HIV. In stable networks of long-term concurrent partnerships, a newly infected person is virtually guaranteed to repeatedly expose another partner during this period, maximizing their chance of transmitting; those partners will in turn expose their other steady partner(s) when their viral load is high, and so on. This can potentially sustain a wave of high viral load as HIV spreads through the network. For all of these reasons, we would expect long-term concurrency to generate higher prevalence than an equivalent number of short term concurrencies. However, few studies have collected the data needed to measure these differences in the patterns of concurrency.

Here we present the results of three in-depth studies of heterosexual behavior in Rakai District in southwestern Uganda, three provinces in Thailand (Bangkok, Udon Thani and Saraburi) and the United States carried out between 1992 and 1994 that permit a detailed comparison of concurrency patterns. All three studies obtained information not only on numbers of sexual partnerships and the prevalence of partnership concurrency, but also on the duration of overlap of concurrent relationships and coital frequency with each concurrent partner. At the time these surveys were carried out, HIV prevalence among heterosexual adults was 18% in Rakai, 2% in Thailand and well below 1% in the US. It is generally believed that prevalence peaked in Uganda in the early 1990s (around the time this study was conducted) and in Thailand around 1993-4 (at the time of this study). In both of these countries, prevalence has since fallen dramatically from the peak. The epidemic in the US was classified as “concentrated” at the time of that study, primarily among men who have sex with men, and injection drug users. Significant racial disparities were beginning to emerge, however, and 1994 was the first year in which non-Hispanic Blacks and Hispanics comprised a majority of both male and female AIDS cases [33], paralleling the longstanding disparities in other sexually transmitted infections [34].

These data sets provide a unique empirical resource; shedding light on the historical variations in sexual behavior that structured the underlying transmission networks leading to three very different epidemic trajectories. The patterns of concurrency they display – defined across multiple measures – are distinct, and consistent with the epidemics that unfolded in each population.

## Results

### Lifetime Partner Numbers

**Figure 1:**
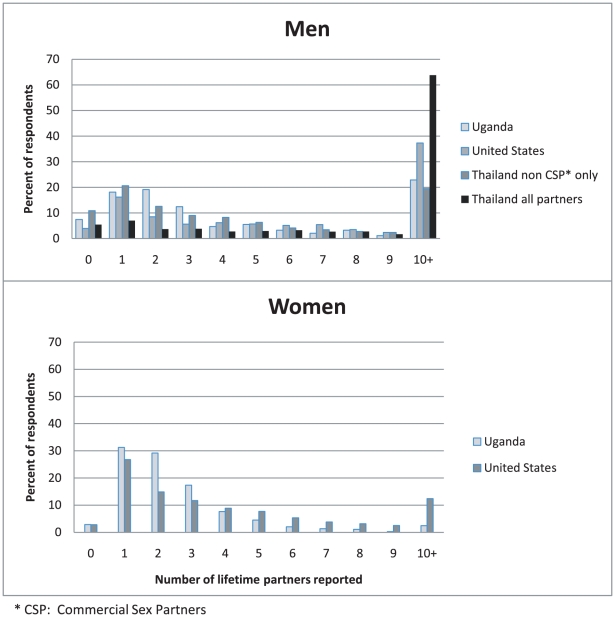
Distributions of the reported lifetime number of partners by country and sex.

Figure 1 shows the distribution of lifetime sexual partnerships for men and women in the three study populations. We did not find large differences in lifetime partners by sampling stratum within populations, so we do not break them out here. (See Methods for explanation of the strata). Lifetime number of partners, like HIV prevalence, is a cumulative measure. If lifetime partner numbers explained the variations in prevalence among these populations, we would expect Uganda to have the greatest density in the upper tail of the distribution, and Thailand and the US to have much lower density there, with the US lowest of all. However, this is not what we find: the fraction of men reporting 10 or more partners is 64% in Thailand, 37% in the US, and 23% in Uganda Among women the fractions are much lower, but US women were more likely to report 10 or more partners (12%) than were Ugandan women (2.5%). Note that in both Uganda and the US, men are more likely than women to report 0 partners.

The reason so many Thai men reported 10 or more lifetime partners is that rates of commercial sex activity are high in this population: over half of the Thai men reported ten or more commercial sex partners during their lifetimes, while only 20% reported ten or more non-commercial partners. The distribution of non-commercial partners for Thai men is also shown in Figure 1, and it is much closer to the US and Ugandan distributions.

### Concurrency

Table 1 presents data on concurrency: summary statistics for the 3 prevalence measures, the overlap duration, and coital frequency in the three countries. Here we found large differences by sampling stratum, so we broke down the estimates accordingly. The US data were broken down by race/ethnicity, as several studies suggest that concurrency may be part of the explanation for disparities in HIV and STI prevalence in the US.

**Table 1:** Concurrency prevalence, overlap duration and coital frequency.

				Prevalence (%)			Overlap (Months)		Median Coital Acts^2^	
			N^1^	Cumulative (last 3 p)	Cumulative (last 3 p last yr)	Point (day of interview)	Mean	Median	More frequent partner	Less frequent partner^3^
Uganda	Men	Trading Center	117	55.3	32.4	16.7	58.8	26	96	14 (52)
		Intermediate	108	57.0	29.5	16.8	63.7	21	90	35 (60)
		Rural	346	55.7	27.1	13.1	65.1	22	85	20 (40)
	Women	Trading Center	161	24.5	4.3	2.3	101.0	123	28	10 (22)
		Intermediate	259	19.2	6.2	0.7	24.5	6	86	11 (66)
		Rural	1003	12.5	2.7	1.3	27.5	36	90	20 (90)
Thailand	Men	Low income	933	52.2	24.3	0.9	8.8	0.1	51+	1 (2–5)
		Trucker	299	71.7	37.5	4.1	23.9	0.1	21–50	1 (1)
United States	Men	White	866	-	10.7	3.1	24.9	4	10+	2–10 (2–10)
		Black	164	-	22.7	11.3	19.3	7	10+	2–10 (2–10)
		Hispanic	109	-	18.9	8.9	29.6	5	10+	2–10 (2–10)
		Other	43	-	8.6	5.2	*	*	*	*
	Women	White	1003	-	5.2	1.3	16.5	4	10+	2–10 (2–10)
		Black	259	-	9.7	3.7	25.9	7	10+	2–10 (10+)
		Hispanic	161	-	7.7	4.9	7.5	5	2–10	2–10 (2–10)
		Other	53	-	0.0	0.0	*	*	*	*

*Too few observations to estimate.

1 Unweighted.

2 In the last year for Uganda and the US, in the last 6 months for Thailand; data for Thailand and US are collected in categories.

3 Median (75^th^ percentile).

Using the cumulative prevalence measures, both Thai and Ugandan men had very high rates of concurrency. Over 50% of the men in each sampling stratum reported at least one concurrency during the last three partnerships, and among Thai truckers the prevalence was over 70%. Restricting the analysis to the last year, the figures dropped by about half for each group, but were still high. Well over 20% of men in each stratum reported a concurrency among the last three partners in the last year, rising above 30% for both Ugandan men living in trading centers and Thai truckers. There were also large differences in the annual cumulative prevalence of concurrency among US men in different race/ethnic groups. About 20% of Black and Hispanic men reported concurrencies in the past year, only slightly lower than the fraction of Ugandan and Thai men who did so. About half as many US white men (11%) and even fewer men of other races (8.6%) did so. Among women, cumulative prevalence of concurrency over the last three partners was available only from Uganda, where we found a strong gradient by stratum. Rural women had the lowest cumulative prevalence of concurrency (12%) and trading center women had the highest (25%). For annual cumulative prevalence, the Ugandan women's figures drop proportionately more than Ugandan men's, with a low of 2.7% among rural women, and a high of 6.2% for women in intermediate-sized villages. The annual cumulative prevalence of concurrency among US women was 9.7% for Black women, 7.7 for Hispanic women, 5.2% for White women, and 0% for all other races.

These findings document both international and within-country variations in the measures of cumulative prevalence of concurrency, and show that they line up with the HIV prevalence differentials within the US and between the US and the other two countries. But the cumulative prevalence measures fail to distinguish Uganda and Thailand. By contrast, the measures of concurrency that capture the impact of long term overlaps – point prevalence, overlap duration, and coital frequency – showed consistent differences across all of these populations that lined up with all of the observed HIV prevalence differentials.

More than 10% of Ugandan men in each stratum had more than one active partnership on the day of the interview, compared to 1% of Thai low income men and 4% of Thai truckers. Among US men, the point prevalence differed by race, hovering around 10% for Black and Hispanic men, compared to 3–5% for Whites and other races. Among women, the point prevalence was very low for Ugandan women in all strata (0.7–2.3%), and for US whites (1.3%), and somewhat higher for US Black and Hispanic women (3.7–4.9%).

The difference between the cumulative and point prevalence measures is due to overlap duration. When this duration is short, we are less likely to observe a concurrent partnership on the day of interview (point prevalence), though we will still capture it with retrospective questions (cumulative prevalence). When we consider overlap durations directly, the patterns are similar to point prevalence. These duration distributions are highly skewed, so we focus on the median as a more stable measure for comparing general tendencies and differences among populations.

The median overlap for Ugandan men and women who report concurrent partners is on the order of 2 years. The one exception is Ugandan rural women, who report median overlaps of only 6 months. This is much longer than both the Thai men's concurrencies (median 1 day), and those of all US subgroups (median 4–9 months).

The pattern of coital acts with each concurrent partner also differs by country. In Uganda and Thailand, the number of acts with the more frequent partner typically ranges from 80–100 over the course of the year (recall that the Thai figures are for 6 months). The one exception is Ugandan trading center women, who report a median of 28 acts per year with their more frequent partner. In the US, the categories make it impossible to observe the upper tail of the distribution in any detail, but all groups report in the top category of 10+ acts per year with their most frequent partner. Thus, most people in all three countries report that the number of sex acts with their most frequent partner is at the high end of the measurement scales. But there are dramatic differences in reports of coital frequency with the less frequent partner. In Uganda, the median ranges from 10–35 acts last year across sex and strata, while the 75^th^ percentile ranges from 22–90 acts. By contrast, in Thailand both the median and 75^th^ percentile are 1 act in the last six months, except for low income Thai men, for whom the 75^th^ percentile is 2–5 acts. In the US, every group reports a median of 2–10 acts last year with their less frequent partner, and for all but one group, this is also the 75^th^ percentile.

Thus, while US men and women accumulated more partners over a lifetime than Ugandans, and Thai men were as likely to engage in concurrent relationships as Ugandan men, the network of concurrent sexual relationships in Uganda was much more stable. In Uganda, having many partners was neither common, nor did it appear to be a primary driver of individual exposure or epidemic potential. However, the Ugandan network had a higher prevalence of concurrency on any given day, much longer overlaps among concurrent partners, and many more coital acts with secondary partners. This kind of stable connectivity, with frequent repeated exposures among all partners, is highly conducive to the spread of HIV and other STIs. It has the potential to place large numbers of people, including those with few partners or even only one, in the path of infection.

## Discussion

This empirical comparison of sexual behavior in the early 1990s in Uganda, Thailand and the US supports the hypothesis that concurrency played an important role in generating the very different trajectories of the AIDS epidemic in these countries, and in the racial and ethnic disparities in HIV prevalence within the US. As in previous studies, we find that differences in the numbers of partners are not consistent with the differences in HIV prevalence [35], [36]. Thai men and American men and women reported considerably more lifetime sexual partners than did their Ugandan counterparts, but HIV prevalence in Uganda was more than ten times higher at its peak than it was in the two other countries. By contrast, variations in the patterns of concurrency do appear to be important indicators of epidemic potential. While Thai men and some groups in the US were as likely as Ugandans to report at least one concurrent partnership during the past year, the point prevalence of concurrency among Ugandan men was 4–16 times higher than among Thai men, and 4–5 times higher than among white men in the US. Ugandan concurrencies were of much longer duration – a median of about two years – than either the Thai (1 day) or US concurrencies (4–9 months), and involved 5–10 times greater intensity of coital risk exposure with the less frequent partner. In the US, Blacks and, to a lesser extent, Hispanics reported higher prevalence, longer overlap duration and greater coital exposure with the less frequent partner than did Whites. Together, the differences in the point prevalence, duration and coital exposure of concurrent partnerships we observe line up perfectly with the HIV prevalence differentials seen in these populations at the time they were measured.

### Historical Data and Subsequent Trends

While these data provide a unique window into the conditions that may have fueled the initial epidemic growth in these countries, they also have interesting implications for interpreting what happened subsequently.

In Uganda, there is good evidence that by 1994, rates of risky sexual behavior had already declined. Two population based studies, from the WHO Global Program on AIDS and the Demographic and Health Surveys, document partner reduction between 1989 and 1995 [37], [38]. This is likely due in part to behavior change – in response to the visible rise of AIDS-related morbidity and mortality, the start of an innovative public health campaign around the message of “Zero Grazing”, and the stabilization that followed the end of the civil war. But differential mortality likely also played a role. Cohort studies documented the high rates of mortality among the HIV infected in this region of Uganda during these years [39], [40], and it is reasonable to assume that initial waves of infection and mortality would have been concentrated among those with higher levels of behavioral risk. Thus the behaviors we observe for Uganda here might be more conservative than those that generated the fast growing epidemic in the early 1980s.

The studies that documented partner reduction in Uganda did not distinguish between changes in concurrency and partner reduction. However, in the study used in this paper, 85% of those who reported 2 or more partners in the last year also reported a concurrency in the last year, suggesting partner reduction in this population could well be a proxy for concurrency reduction. If so, this would strengthen our conclusion that long-term concurrency in Uganda played a key role generating the epidemic, and that reductions in concurrency contributed to the rapid decline in HIV prevalence after 1991. This would be consistent with empirical evidence from Manicaland, Zimbabwe that the HIV prevalence decline during the early 2000's coincided with a marked decline in concurrent partnerships [41], [42].

In Thailand, commercial sex patronization by men dropped nearly 50% from 1993 to 1996, and condom use among those who did report commercial sex peaked at around 90% during this period [43]. The same study found no changes in sexual behavior among single or married women in the general population. The subsequent decline in HIV prevalence among Thai men is well known. What is less well known is that prevalence also declined dramatically among young women: from 1995 to 2003, prevalence decreased by 75% among pregnant women under 20 (2.4% to 0.8%) and by 48% among 20–24 year olds (2.5% to 1.4%). Prevalence changes among young women are often used as a proxy for changes in incidence. Almost all of the concurrency observed in the Thai study described here was related to commercial sex. This strongly suggests that the decline in commercial sex led to a concomitant decline in concurrency, and an immediate decline in HIV incidence.

In the US, subsequent studies have documented a continuing racial disparity in concurrency, among both heterosexuals [11], [44], [45] and MSM [46]. The evidence for racial disparities in HIV prevalence has also continued to accumulate [47], [48]. In Washington, DC, the HIV prevalence among African Americans has now reached 3% [49]. In a comprehensive analysis of a large national population-based study that collected both biomarkers for HIV and 3 other STIs, and a wide array of behavioral measures, the racial disparities in HIV and other STI could not be explained by any of the traditional measures of individual sexual or drug-related risk [50]. In a simulation study based on the same data, the concurrency differences were found to explain nearly two-thirds of the racial difference in epidemic potential [11].

### Study Limitations

The analyses presented here are ecological in nature, in that they examine associations between population HIV prevalence trajectories and the rates of reported concurrency. As the effect of concurrency is a population level effect – increased network connectivity – ecological analysis is an appropriate method to use here. But, as noted above, we only observe concurrency behavior at one time point, which obscures at least part of the temporal relationship between concurrency and HIV prevalence. Several issues of sampling and measurement should also be kept in mind when interpreting these results. First, women from the general population were not included in the Thai sample. Other studies at the time show that very few women reported more than one partner in the last year [43], so we can assume the rates of concurrency are very low. This would imply a strong gender asymmetry in the cumulative prevalence of concurrency, as also observed in both Uganda and the US. For the purposes here, the absence of data for Thai women is not likely to change the implications of our findings, or our conclusions. However, since the connectivity in heterosexual networks depend upon women's behavior as well as men's, data on women are necessary to develop accurate models of the population spread of HIV. Second, the lack of sampling weights in the Thai and Ugandan studies makes it inappropriate to conduct tests of statistical significance. The key differences we observe here are not subtle however, and therefore the contrasting patterns are likely to be valid, even if the exact differences in magnitude are not. Finally, differences in the coital frequency questionnaires makes cross-country comparisons less precise, but again, the differences we see here are very large, even with the blunt measures used in the US study. More comparable and precise measures would be preferable, but are unlikely to affect our conclusions.

### Implications for Data Collection

This study has a number of implications for data collection. First, the simple nine question approach to measuring concurrency (dates of first and last sex, and status of relationship on the day of interview, for the most recent three partners) provides a detailed picture of variations in patterns of concurrency. The information that can be extracted from these data allow for a detailed comparison of point prevalence, cumulative prevalence and overlap duration, key factors that influence the impact concurrency has on HIV transmission. The additional questions on coital frequency provide information that is important for accurate modeling of the transmission dynamic impact. Unfortunately, many recent surveys that use an egocentric network module to measure concurrency, including the Demographic and Health Surveys, fail to ask even the three basic questions – typically missing either the active status of the relationship on the day of interview or the date of last sex. This seriously compromises estimates of concurrency within surveys, and prevents comparison of concurrency prevalence across surveys. The nine question module, which was recently endorsed by the UNAIDS reference group [51] should be regarded as the minimum required for measuring concurrency in empirical studies.

Second, point prevalence of concurrency is clearly a better indicator of epidemic potential than the more commonly used measures of cumulative prevalence or annual cumulative prevalence of concurrency. Using either of the cumulative prevalence measures, we would have predicted that the epidemic in Thailand would have followed the course of the epidemic in Uganda. Point prevalence was a far better predictor of the epidemic trajectories we observed, because it captures overlap duration and, by implication, some element of coital frequency. Short overlap concurrencies are unlikely to be observed on any single day, and the shorter the overlap, the fewer the coital acts that provide the bidirectional exposure that enhances the epidemic potential of concurrency.

Finally, while point prevalence is the UNAIDS reference group's recommended indicator for measuring concurrency, we note that there is some disagreement about how point prevalence should be measured. UNAIDS currently recommends that researchers ask about the start and end dates of a person's last three partnerships during the past year, and then calculate the number of ongoing partnerships the person had exactly six months before the date of the interview [51]. This measure has several sources of error: uncertainty due to date precision (dates are often collected as month/year, ignoring days), missing dates (1 for each date, or 4 total), uncertainty associated with retrospective reporting (4 again), and truncation bias (if the most recent four partners are within the 6 month retrospective window). The amount of uncertainty and error in the resulting estimate can be substantial. Calculating this indicator is also technically challenging, even if the primary data are collected correctly.

Instead, we suggest using point prevalence measured on the day of the interview, as in the studies discussed here. This requires three simple questions: for each partner, the respondent is asked “Are you still sexually active with this partner?” or “Do you expect to have sex with this partner again?” While this raises reasonable concerns about the validity and reliability of “intention” questions – after all, who can predict the future? – the potential for measurement error is still likely to be lower than the “six months ago” concurrency indicator.

Some evidence for the internal validity of the day of interview point prevalence indicator can be found in the data itself. A classic diagnostic for under- or over-reporting on sexual behavior surveys is the discrepancy between men's and women's reports of the number of partners. The retrospective window is an important determinant of the size of these discrepancies. In the US, the discrepancy drops from a ratio of 4∶1 using the lifetime partner reports, to 1.5∶1 using partners in the last year [52], and effectively 1∶1 using the number of partnerships ongoing on the date of interview [53]. This suggests both that active status can be reliably reported, and that point prevalence estimates, when they are based on the shortest possible recall period, will be more reliable than cumulative prevalence estimates. All of this lends further support to UNAIDS's recommendation to use point prevalence as the primary indicator of concurrency. However, the point to measure should be the day of the interview, rather than six months earlier.

The results presented here underscore the importance of understanding sexual networks in detail, beyond superficial measures of multiple partnerships, or even prevalence of concurrency. While this might seem like a complex empirical task, in fact only 9 questions are required to assess point prevalence and overlap duration. During the past 20 years, these questions have been used in many types of surveys and a wide range of populations. Collecting this information is no more difficult than collecting data on sexual behavior in general. Given the potential value of the data these questions produce, it would be useful to focus some effort on assessing their reliability and validity.

### Implications for HIV Prevention Strategy

Unlike infectious diseases that spread through vectors, point sources and casual interaction, HIV and other sexually transmitted diseases spread via dyadic relationships. The implications for HIV prevention are that those typically considered at “low risk”, including those with only one partner, may be at high risk if they are linked through that partner to just one other person who is in turn linked to a larger sexual network. This limits the utility of campaigns that stress individual behavior change as a strategy for avoiding infection. The behaviors that gave rise to the HIV epidemics in Thailand, Uganda and among US gay men were very different. However, in all three places, indigenous, locally developed strategies to spread awareness about risky sexual behavior—whether concurrent, casual or commercial–and confront sexual norms, in a pragmatic, non-moralistic manner helped reduce HIV transmission. International public health agencies have a responsibility to support local efforts by providing people with accurate information about how HIV is spreading in their communities, and in particular, how long term concurrency can connect people into a large network, raising the risk of infection for everyone, including those with only a few trusted, long-term partners.

## Materials and Methods

The data for this analysis comes from three behavioral surveys conducted in the early 1990s in Uganda, Thailand, and the United States.

### Study populations

#### Uganda

The Rakai-Project Sexual Network Study was conducted during 1993–1994. It used a cross-sectional study design with a random sample of 1627 adults aged 15–49 years in the Rakai District of rural southwest Uganda. The sample was stratified by residence in a trading center, an intermediate sized town or a village because this is known to influence HIV risk. [Serwadda et al. 1992] The survey response rate was 92%. More details can be found in [54, ch. 2].

#### United States

The National Health and Social Life Survey (NHSLS) was conducted in 1992. It used a cross-sectional study design with a nationally representative stratified random sample of 3,432 American women and men between the ages of 18 and 59. The survey response rate was 82%. More details can be found in Laumann [55].

#### Thailand

The Behavioral Research on AIDS in Thailand Survey (BRAIDS) was conducted during 1992 in one urban (Bangkok) and two semi-urban (Udon Thani and Saraburi) provinces in northeastern and central Thailand. It used a cross-sectional study design with a quota sample of 1075 low-income men aged 17–45 years, and a subsample of a high-risk population of 330 male long-haul truck drivers aged 14–62. Women in the general population were not interviewed. More details can be found in [54, ch. 2].

For comparability across study populations, we restrict the analysis to respondents aged 18–45.

### Measures and methods

The questionnaires used in each survey included a “local network module” for eliciting partner-specific information about sexual behavior [54]. The Ugandan and Thai studies collected information about each respondent's three most recent sexual partners. The US study collected information about all partners in the last year.

For comparability across studies, we use comparable data on the three most recent partners in the last year.

All three studies collected the dates of first and last sex with each of the three last partners, in the past year. If a respondent reported that s/he expected to have sex with a particular partner again, or if s/he was currently cohabiting with this partner, the sexual relationship was considered to be ongoing. These dates defined the partnership intervals, which were then checked for overlap across all three potential partnership pairs (partners 1 and 2, 1 and 3, and 2 and 3). If the start date of a more recent partnership preceded the end date of an earlier partnership, the partnerships were defined as concurrent, and the duration of overlap was defined as the difference between these two dates.

We report three measures of concurrency prevalence: 1) cumulative prevalence is the fraction of people reporting that at least two of their three most recent partnerships overlapped (only available for the Uganda and Thailand surveys); 2) annual cumulative prevalence is the fraction reporting that during the past year, two or more of their last three partners overlapped; and 3) point prevalence is the fraction who had more than one ongoing sexual partnership on the day of the interview.

We report the mean and median overlap duration of all concurrent partnerships. Only respondents with concurrent partnerships contribute observations, and those reporting more than one concurrency contribute as many observations. Ongoing partnerships are treated as censored observations, with durations estimated using Kaplan-Meier methods.

We also report data on the number of coital acts with each concurrent partner. In the Ugandan study, the number of acts in the last year was directly reported for each partnership. In the Thai study, the questions referred to the last six months only, and respondents were asked to choose from the following categories: only once, 2–5x, 6–10x, 11–20x, 21–50x, 50+. In the US study, the questions referred to the last year, and respondents were asked to choose from a broader set of categories: only once, 2–10x, 10+. Using concurrent partnerships as the unit of analysis, we report the median number of acts reported (or the median category) for the most frequent partner, and both the median and the 75^th^ percentile for the less frequent partner.

The use of statistical tests here is not strictly appropriate, as the sampling strategies varied across the studies: the US is the only study with sample weights, while the Thai and Ugandan samples are stratified but not weighted. We report the concurrency findings by sampling stratum for Uganda and Thailand to adjust for the study designs in the absence of sample weights, and use the sample weights for the US estimates. While we do not test for statistical significance, the key differences observed among the study populations are very large, allowing substantive inferences to be drawn.
